# Impact of bioprosthetic valve type on peri-procedural myocardial injury and mortality after transcatheter aortic valve replacement

**DOI:** 10.1007/s00380-021-01861-8

**Published:** 2021-05-07

**Authors:** Vincenzo De Marzo, Gabriele Crimi, Matteo Vercellino, Stefano Benenati, Fabio Pescetelli, Roberta Della Bona, Matteo Sarocchi, Marco Canepa, Manrico Balbi, Italo Porto

**Affiliations:** 1 DICATOV–Cardiothoracic and Vascular Department, San Martino Hospital, IRCCS for Oncology and Neurosciences, Genoa, Italy; 2 Department of Internal Medicine and Specialties (DIMI), Clinic of Cardiovascular Diseases, University of Genoa, Viale Benedetto XV, 10, 16132 Genoa, Italy

**Keywords:** Transcatheter aortic valve replacement, Heart valve prosthesis, Troponin, Heart injuries, Creatine kinase MB form, Troponin I

## Abstract

Peri-procedural myocardial injury (PPMI) is a common complication after transcatheter valve replacement (TAVR), often remaining clinically silent. The role of valve type on PPMI and the association between PPMI and mortality are still unclear. We sought to evaluate predictors and outcome of PPMI after TAVR, and the impact of self-expandable valve (SEV) vs. balloon-expandable valve (BEV) deployment on PPMI. Consecutive patients who underwent successful TAVR in a single-center from January 2014 to December 2019 were included. PPMI was defined according to a modified Valve Academic Research Consortium (VARC)-2 definition as a post-procedure elevation of troponin (with a peak value ≥ 15-times the upper-reference limit) < 72 h after TAVR. We included 596 patients, of whom 258 (43.3%) were men. Mean age was 83.4 ± 5.5 years. We deployed 368 (61.7%) BEV and 228 (38.3%) SEV. PPMI was observed in 471 (79.0%) patients. At multivariable analysis, SEV (OR 2.70, 95% CI 1.64–4.55, *p* < 0.001), creatinine clearance (OR 0.98, 95% CI 0.97–1.00, *p* = 0.011), and baseline ejection fraction (OR 1.05, 95% CI 1.02–1.07, *p* < 0.001) were independent predictors of PPMI; these findings were also confirmed using a propensity-weighted analysis. Thirty-day and 1-year all-cause mortality rates were 2.5% and 8.1%, respectively. No associations between PPMI and 30-day (*p *= 0.488) or 1-year (*p* = 0.139) all-cause mortality were found. Independent predictors of 30-day mortality were increasing EUROSCORE II (HR 1.16 per score point, 95% CI 1.08–1.19, *p* < 0.001) and life-threatening/major bleeding complications (HR 5.87, 95% CI 1.28–26.58, *p* = 0.019), whereas EUROSCORE II (HR 1.08, 95% CI 1.04–1.13, *p* = 0.031) and acute kidney injury (HR 2.59, 95% CI 1.20–5.35, *p* = 0.020) were related to 1-year mortality. PPMI is frequent after TAVR, but it does not affect 30-day or 1-year all-cause mortality. SEV implantation is associated with an increased frequency of PPMI.

## Introduction

Transcatheter aortic valve replacement (TAVR) is the treatment of choice for severe aortic stenosis (AS) at high risk for conventional surgery, and it is increasingly being performed in patients at intermediate and low risk [1–4]. As the TAVR community moves towards treating less complex patients, recognition and management of even minor intra- and peri-procedural complications becomes pivotal.

Among complications of TAVR, peri-procedural myocardial injury (PPMI) is common [1–4], occurring in roughly half of procedures if a sensitive definition is used [1, 5–13]. The etiopathogenesis of PPMI after TAVR (PPMI-TAVR) is multifactorial, depending on both patient-related factors, such as aortic atheroma burden, as well as procedural factors, such as pre-TAVR balloon valvuloplasty, rapid pacing, and valve post-dilatation [1, 8, 10–13]. In this regard, there might be an increased risk of PPMI-TAVR if a self-expandable valve (SEV), as compared to a balloon-expandable valve (BEV), is chosen. Indeed, the profoundly different valve designs and implantation techniques may affect procedural time, and the tissue compression by valve frames is clearly different.

The impact of PPMI on prognosis, however, remains controversial [2, 14–17], as most data derive from observational studies applying different PPMI definitions to heterogeneous populations.

Therefore, the primary aim of our retrospective study was to determine the incidence, predictors and impact of SEV (as compared to BEV) deployment on PPMI; the secondary aim was to explore the effect of PPMI (and of SEV vs. BEV) on clinical outcomes.

## Materials and methods

### Study population

This is a retrospective study using a prospectively maintained database. All consecutive patients with symptomatic severe AS who had undergone TAVR between January 2014 and December 2019 at our institution were included. Therefore, we excluded peri-procedural deaths and patients with incorrect positioning of valve for improper anatomical location or use of > 1 valve.

All patients signed an informed consent allowing the utilization of their anonymized clinical information for medical research purposes, as approved by the local Institutional Review Board (Genova TAVR registry, N° Registro CER Liguria: 331/2020—DB id 10,646).

### Demographic and clinical data

For every patient, we collected gender, age, body mass index (BMI), New York Heart Association (NYHA) class, diabetes mellitus (DM), chronic kidney disease (CKD), chronic obstructive pulmonary disease (COPD), history of neurological disease, coronary artery disease (CAD), previous acute coronary syndrome (irrespective of myocardial revascularization), atrial fibrillation (AF), prior valvuloplasty, and European System for Cardiac Operative Risk Evaluation score II (EUROSCORE II).

CAD was defined as the presence of at least one coronary stenosis ≥ 50% in vessels ≥ 1.5 mm. Echocardiography data, e.g., left ventricular ejection fraction (LVEF), maximum and mean aortic valve gradient were also collected.

### Laboratory values and measurements

Among biomarkers, creatinine, creatinine clearance (CrCl) estimated by Cockcroft–Gault formula, and troponin I (TnI) were assessed daily until hospital discharge.

Plasma TnI concentration was measured using a sandwich chemiluminescent immunoassay based on LOCI^®^ technology on Dimension Vista^®^ 1500 System. The limit of quantitation (functional sensitivity), which corresponds to the TnI concentration at which the coefficient of variation is 10%, was < 0.04 µg/L [18]. The upper-reference limit (URL), as defined at the 99th percentile of the reference interval, was 0.046 µg/L.

### Procedural data

TAVR-related data such as procedural time, type of vascular access, type of valve, valve-in-valve implantation, pre- and post-TAVR dilatation, and the use of rapid pacing were recorded.

Successful TAVR were defined according to the presence of all the following criteria: absence of procedural mortality; correct positioning of a single prosthetic heart valve into the proper anatomical location; intended performance of the prosthetic heart valve (no prosthesis-patient mismatch and mean aortic valve gradient < 20 mmHg or peak velocity < 3 m/s) [19].

For the purpose of this analysis, we included the following SEV: CoreValve (Medtronic, Minneapolis, Minnesota), Evolut R (Medtronic, Minneapolis, Minnesota), Evolut PRO (Medtronic, Minneapolis, Minnesota), Symetis ACURATE neo (Symetis/Boston, Ecublens, Switzerland) and NVT ALLEGRA (New Valve Technology [NVT], Hechingen, Germany). SAPIEN XT (Edwards Lifesciences, Irvine, California), SAPIEN 3 (Edwards Lifesciences, Irvine, California), and SAPIEN 3 ULTRA (Edwards Lifesciences, Irvine, California) were considered BEV.

### VARC-2 definitions

Length of hospital stay, VARC-2 outcomes (PPMI, AKI, stroke, vascular complications, bleedings, permanent pacemaker implantation, cardiac tamponade), and mortality at 30-day and 1-year follow-up were collected.

PPMI was defined according to a modified Valve Academic Research Consortium (VARC)-2 criteria as a post-procedure elevation of troponin (with a peak value exceeding 15 × as the upper-reference limit) within 72 h after TAVR, at least in one sample. If troponin was increased at baseline (> 99th percentile), a further post-procedural increase of at least 50% was required to meet the endpoint definition [19]. Since ischemic symptoms in the peri-procedural setting appear misleading and confounding in nature, we decided to focus only on the laboratory markers (e.g., troponin elevation) to define PPMI.

AKI was defined as an increase of at least 0.3 mg/dL in serum creatinine or a urine output worsening (< 0.5 mL/kg for < 12 h) occurring within 7 days after the procedure [19].

### Statistics

Categorical variables were expressed as frequencies and percentages and compared by Chi-square test or Fisher’s exact-test; continuous variables were reported as mean and relative standard deviation (SD) or median and interquartile range (IQR) and were compared using the unpaired Student *t* test or the Wilcoxon rank-sum test, depending on the variable distribution.

Univariate logistic regression analysis was used to identify clinical and procedural factors associated with PPMI. Thereafter, a multivariate logistic regression model was fitted, including all variables with *p* values < 0.10 in the univariate analysis, plus age and sex as background variables, to explore the independent impact of those variables on the development of PPMI.

Univariate survival analysis was performed fitting Kaplan–Meier curves (time to all-cause death at 30-day and 1-year) for patients developing or not PPMI. Subsequently, uni- and multivariate Cox models (for 30-day mortality and 1-year mortality) were constructed to estimate the hazard ratios (HRs) and 95% confidence interval (CI) of baseline, procedural characteristics, and VARC-2 outcomes on 30-day and 1-year mortality. The variables were selected on a statistical and clinical basis. In particular, laboratory and procedural variables with a *p* < 0.10 association with the outcome of interest were included (avoiding collinearity), and clinically meaningful predictors were also assessed based on an updated literature search [19].

Sensitivity analysis using multiple imputation with predictive mean matching and the exclusion of the covariates with missingness were used.

Finally, to better investigate the role of type of vascular access, we repeated the statistical analysis for PPMI and 30-day/1-year mortality excluding those patients who received transapical access due to its higher invasiveness.

A two-tailed *p* value of 0.05 was considered to reject the null hypothesis. All analyses were performed with R environment 3.6.3 (R Foundation for Statistical Computing, Vienna, Austria) using *tableone*, *finalfit*, *survival*, and *twang* packages [20].

## Results

We included 695 patients who underwent TAVR at our institution from January 2014 to December 2019. Of those, 23 (3.3%) patients were excluded due to unsuccessful TAVR (11 for peri-procedural deaths, 12 for incorrect positioning of valve for improper anatomical location or use of > 1 valve) and 76 (10.9%) due to the lack of appropriate biomarkers measurements before or during hospital stay, leading to a final study cohort of 596 patients. Baseline clinical features are outlined in Table 1. Mean age was 83.4 ± 5.5 years and 258 (43.3%) of patients were men, median EUROSCORE II was 4.1% (IQR 3.3–6.5). Two-hundred sixty-seven (44.8%) patients had CAD, baseline median creatinine was 1.1 (IQR 0.9–1.4) mg/dL, whereas baseline median CrCl was 37.5 (IQR 28.6–49.6) mL/min.

**Table 1:** Baseline patient’s characteristics. All measures expressed as *n* (%), mean (SD) or median with interquartile range (quartile 1 to quartile 3)

Variable	Overall (*n* = 596)	No PPMI (*n* = 125)	PPMI (*n* = 471)	*p* value
Age	83.4 (5.5)	83.1 (5.7)	83.5 (5.4)	0.420
Men	258 (43.3)	68 (54.4)	190 (40.3)	0.007
BMI	25.3 (4.2)	25.6 (4.4)	25.2 (4.1)	0.274
NYHA				
Class I	31 (5.2)	7 (5.6)	24 (5.1)	0.012
Class II	246 (41.3)	44 (35.2)	202 (42.9)	
Class III	280 (47.0)	58 (46.4)	222 (47.1)	
Class IV	39 (6.5)	16 (12.8)	23 (4.9)	
EUROSCORE II	4.4 (3.3–6.5)	4.6 (3.4–7.0)	4.3 (3.2–6.4)	0.194
Diabetes	166 (27.9)	47 (37.6)	119 (25.3)	0.012
Previous acute coronary syndrome	108 (18.1)	29 (23.2)	79 (16.8)	0.129
CAD	267 (44.8)	70 (56.0)	197 (41.8)	0.006
Previous valvuloplasty	52 (8.7)	16 (12.8)	36 (7.6)	0.097
Pre-TAVR creatinine (mg/dL)	1.1 (0.9–1.4)	1.1 (0.9–1.4)	1.1 (0.9–1.4)	0.914
Pre-TAVR CrCl (mL/min)	37.5 (28.6–49.6)	41.3 (32.0–53.8)	36.8 (27.6–48.1)	0.012
Baseline hemoglobin (mg/dL)	12.0 (10.9–13.1)	12.2 (11.5–13.2)	11.9 (10.8–12.9)	0.015
Baseline NT-proBNP (ng/dL)	3401.0 (1564.8–7788.3)	3897.0 (2023.0–8023.0)	3258.0 (1403.5–7672.5)	0.252
Atrial fibrillation	205 (34.4)	47 (37.6)	158 (33.5)	0.466
COPD	141 (23.7)	36 (28.8)	105 (22.3)	0.160
LVEF (%)	55.0 (48.0–60.0)	55.0 (40.0–55.0)	55.0 (50.0–60.0)	< 0.001
Maximum aortic gradient (mmHg)	100.0 (82.0–112.0)	96.0 (69.8–108.3)	99.0 (80.0–112.0)	0.052
Mean aortic gradient (mmHg)	51.5 (44.0–60.0)	49.0 (40.0–60.0)	51.5 (44.8 to 60.0)	0.056

*BMI* body mass index, *BNP* brain natriuretic peptide, *CAD* coronary artery disease, *COPD* chronic obstructive pulmonary disease, *CrCl* creatinine clearance, *LVEF* left ventricular ejection fraction, *NYHA*; new york heart association, *PPMI* post-procedure myocardial injury, *TAVR* transcatheter aortic valve replacement

Median procedural time was 113 (IQR 86.0–146.0) min, 551 (92.4%) procedures were transfemoral, 336 (56.4%) patients underwent pre-dilatation, 57 (9.6%) post-dilatation, whereas rapid pacing was used in 505 (84.7%). Other procedural variables are shown in Table 2.

**Table 2:** Procedural characteristics. All measures expressed as n (%), mean (SD) or median with interquartile range (quartile 1 to quartile 3)

Variable	Overall (*n* = 596)	No PPMI (*n* = 125)	PPMI (*n* = 471)	*p* value
*Valve type*				< 0.001
SAPIEN XT	29 (4.9)	9 (7.2)	20 (4.2)	
SAPIEN 3	285 (47.8)	71 (56.8)	214 (45.4)	
SAPIEN 3 ULTRA	54 (9.1)	17 (13.6)	37 (7.9)	
CoreValve	70 (11.7)	6 (4.8)	64 (13.6)	
Evolut R	58 (9.7)	5 (4.0)	53 (11.3)	
Evolut PRO	85 (14.3)	15 (12.0)	70 (14.9)	
ACURATE neo	10 (1.7)	1 (0.8	9 (1.9)	
NVT ALLEGRA	5 (0.8)	1 (0.8)	4 (0.8)	
*Vascular access*				
Femoral	551 (92.4)	120 (96.0)	436 (92.6)	0.407
Transapical	15 (2.5)	2 (1.6)	13 (2.8)	
Subclavian	30 (5.0)	5 (4.0)	25 (5.3)	
Valve-in-valve	18 (3.0)	5 (4.0)	13 (2.8)	0.670
Pre-dilatation	336 (56.4)	64 (51.2)	272 (57.7)	0.341
Post-dilatation	57 (9.6)	11 (8.8)	46 (9.8)	0.944
Rapid pacing	505 (84.7)	111 (88.8)	394 (83.7)	0.301
Procedural time	113.0 (86.0 to 146.0)	117.0 (87.0 to 140.8)	111.00 (86.0 to 147.0)	0.760

*PPMI* post-procedure myocardial injury

We implanted 29 (4.9%) SAPIEN XT, 285 (47.8%) SAPIEN 3, 54 (9.1%) SAPIEN 3 ULTRA, and 5 (0.8%) NVT ALLEGRA, 70 (11.7%) CoreValve, 58 (9.7%) Evolut R, 85 (14.3%) Evolut PRO, and 10 (1.7%) ACURATE neo.

### PPMI predictors

Incidence of VARC-2 adverse events is presented in Table 3; PPMI was observed in 475 (79.0%) patients.

**Table 3:** VARC-2 outcomes. All measures expressed as *n* (%), mean (SD) or median with interquartile range (quartile 1 to quartile 3)

Variable	Overall (*n* = 596)	No PPMI (*n* = 125)	PPMI (*n* = 471)	*p* value
All-cause 30-day mortality	15 (2.5)	2 (1.6)	13 (2.8)	0.679
All-cause 1-year mortality	48 (8.1)	6 (4.8)	42 (8.9)	0.167
Cerebrovascular complication	15 (2.5)	3 (2.4)	12 (2.5)	1.000
Major stroke	4 (0.7)	1 (0.8)	3 (0.6)	
Minor stroke	11 (1.8)	2 (1.6)	9 (1.9)	
Post-TAVR TnI	1.5 (0.8–2.8)	0.4 (0.2–0.6)	2.0 (1.2–3.3)	< 0.001
Bleeding complication	99 (16.6)	14 (11.2)	85 (18.0)	0.090
Life-threatening	9 (1.5)	1 (0.8)	8 (1.7)	
Major	28 (4.7)	4 (3.2)	24 (5.1)	
Minor	62 (10.4)	9 (7.2)	53 (11.3)	
Vascular complication	90 (15.1)	13 (10.4)	78 (16.6)	0.164
Major	32 (5.4)	3 (2.4)	29 (6.2)	
Minor	59 (9.9)	10 (8.0)	49 (10.4)	
Post-TAVR creatinine (mg/dL)	1.2 (0.9–1.6)	1.2 (1.0–1.5)	1.2 (0.9–1.6)	0.433
Post-TAVR CrCl (ml/minute)	35.3 (25.6–47.4)	40.6 (31.7–54.0)	34.1 (24.1–46.4)	< 0.001
Acute kidney injury	152 (25.5)	26 (20.8)	126 (26.8)	0.214
Permanent PM implantation	84 (14.1)	10 (8.0)	74 (15.7)	0.040
Cardiac tamponade	7 (1.2)	2 (1.6)	5 (1.1)	0.976

*CrCl* creatinine clearance; *PM*: pacemaker; *PPMI*: post-procedure myocardial injury; *TAVR*: transcatheter aortic valve replacement; *TnI* troponin I

Patients who developed PPMI was less frequent male (40.3% vs 54.4%, *p* = 0.007) and had less history of CAD (41.8% vs. 56.0%, *p* = 0.006) or diabetes (25.3% vs. 37.6%, *p* = 0.012); furthermore, patients with PPMI had higher baseline LVEF (55.0 [IQR 50.0–60.0] vs. 55.0 [IQR 40.0–55.0], *p* = 0.001), lower baseline CrCl (36.8 [IQR 27.6–48.1] mL/min vs. 41.3 [IQR 32.0–53.8], *p* = 0.001), and were implanted more frequent with SEV rather than BEV (42.5% vs. 22.4%, *p* < 0.001).

After adjusting for confounders in the multivariable analysis, we found that SEV implantation (OR 2.70, 95% CI 1.64–4.55, *p* < 0.001), lower baseline CrCl (OR 0.98, 95% CI 0.97–1.00, *p* = 0.011), and higher LVEF (OR 1.05, 95% CI 1.02–1.07, *p* < 0.001) were independent predictors of PPMI (Table 4). Notably, SEV, baseline CrCl, and LVEF remained independently associated with higher PPMI even when pre-dilation, post-dilatation, and the use of rapid pacing were forced into the model to account for procedural potential confounders (Table 5).

**Table 4:** Univariate and multivariable logistic regression models for post-TAVR myocardial injury

Predictor	Univariate			Multivariate		
	OR	95% CI	*p* value	OR	95% CI	*p* value
Sex (male)	0.56	0.37–0.82	0.390	0.81	0.50–1.31	0.390
Age	1.01	0.98–1.05	0.466	0.96	0.92–1.01	0.111
SEV	2.52	1.62–4.05	< 0.001	2.70	1.64–4.55	< 0.001
Pre-TAVR CrCl	0.99	0.98–1.00	0.031	0.98	0.97–1.00	0.011
LVEF	1.03	1.01–1.05	< 0.001	1.05	1.02–1.07	< 0.001
Diabetes	0.57	0.38–0.87	0.009	0.82	0.51–1.36	0.443
CAD	0.58	0.39–0.86	0.007	0.67	0.42–1.07	0.090
Maximum aortic gradient	1.01	1.00–1.02	0.014	1.01	1.00–1.02	0.165

*CAD* coronary artery disease, *CrCl* creatinine clearance, *LVEF* left ventricular ejection fraction, *SEV* self-expandable valve, *TAVR* transcatheter aortic valve replacement

**Table 5:** Univariate and multivariable logistic regression models for post-TAVR myocardial injury adjusted for pre-dilatation, post-dilatation, and rapid pacing

Predictor	Univariate			Multivariate		
	OR	95% CI	*p* value	OR	95% CI	*p *value
Sex (male)	0.56	0.37–0.82	0.390	0.76	0.46–1.26	0.283
Age	1.01	0.98–1.05	0.466	0.97	0.92–1.01	0.156
SEV	2.52	1.62–4.05	< 0.001	2.99	1.59–5.99	0.001
Pre-TAVR CrCl	0.99	0.98–1.00	0.031	0.98	0.97–1.00	0.015
LVEF	1.03	1.01–1.05	< 0.001	1.05	1.02–1.07	< 0.001
Diabetes	0.57	0.38–0.87	0.009	0.74	0.45–1.24	0.244
CAD	0.58	0.39–0.86	0.007	0.76	0.46–1.25	0.280
Maximum aortic gradient	1.01	1.00–1.02	0.014	1.01	1.00–1.02	0.178
Pre-dilatation	1.24	0.82–1.86	0.312	1.04	0.63–1.73	0.867
Post-dilatation	1.09	0.56–2.28	0.809	0.87	0.41–1.99	0.729
Rapid pacing	0.65	0.34–1.15	0.158	1.37	0.58–3.24	0.469

*CAD* coronary artery disease, *CrCl* creatinine clearance, *LVEF* left ventricular ejection fraction, *SEV* self-expandable valve, *TAVR* transcatheter aortic valve replacement

**Fig. 1:**
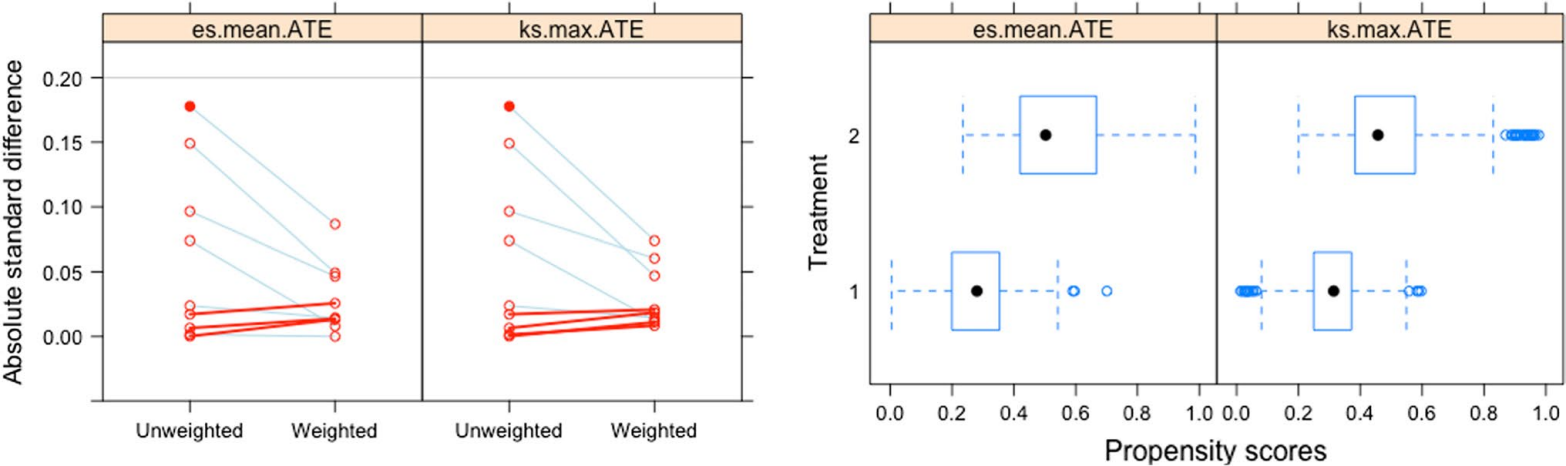
Results of balancing SEV vs. BEV with an average treatment effect propensity score. **a** Absolute standard differences for baseline covariates before and after propensity score showing well balancing of covariates: absolute standard differences for covariates decrease between the two investigational groups (BEV vs. SEV) when propensity score weighting was applied; **b** propensity scores for covariates before and after weighting: after well balancing of covariates, the scores appear more overlapping between the two investigational groups (BEV vs. SEV) with less dispersion at extreme values. *BEV* balloon-expandable valve, *SEV* self-expandable valve

Furthermore, after well balancing SEV vs. BEV with an average treatment effect propensity score (Fig. 1), we confirmed a significant higher incidence of PPMI among patients receiving SEV as compared to BEV (*p *< 0.001).

Finally, SEV implantation, baseline CrCl, and LVEF remained significant when we repeated the regression analysis for PPMI excluding those patients who received transapical access (Table 6).

**Table 6:** Univariate and multivariable logistic regression models for post-TAVR myocardial injury excluding patients who received transapical access

Predictor	Univariate			Multivariate		
	OR	95% CI	*p* value	OR	95% CI	*p* value
Sex (male)	0.56	0.38–0.84	0.005	0.71	0.42–1.18	0.185
Age	1.01	0.97–1.05	0.599	0.97	0.92–1.02	0.276
SEV	2.64	1.68–4.24	< 0.001	3.48	2.01–6.26	< 0.001
Pre-TAVR CrCl	0.99	0.98–1.00	0.031	0.98	0.97–1.00	0.021
LVEF	1.04	1.02–1.06	< 0.001	1.04	1.02–1.07	< 0.001
Diabetes	0.53	0.35–0.81	0.003	0.66	0.40–1.10	0.105
CAD	0.56	0.37–0.84	0.005	0.61	0.37–0.99	0.056
Maximum aortic gradient	1.01	1.00–1.02	0.012	1.01	1.00–1.02	0.185

*CAD* coronary artery disease, *CrCl* creatinine clearance, *LVEF* left ventricular ejection fraction, *SEV* self-expandable valve, *TAVR* transcatheter aortic valve replacement

### All-cause mortality

**Fig. 2:**
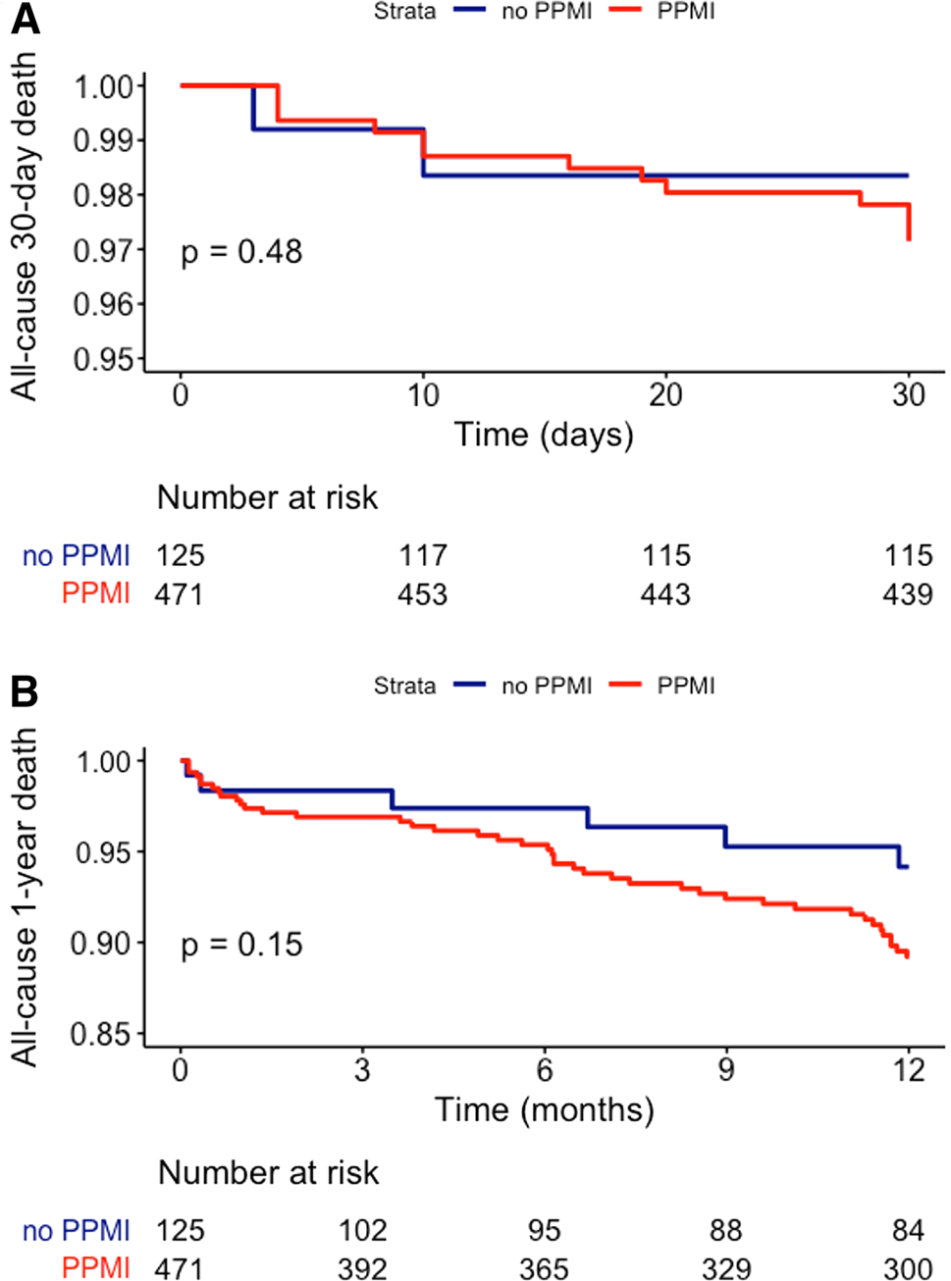
Kaplan–Meier curves for 30-day (panel A) and 1-year (panel B) all-cause mortality according to PPMI. *PPMI* post-procedure myocardial injury

Overall, at a median follow-up of 18.5 (IQR 7.6–34.0) months, we observed 15 (2.5%) 30-day and 48 (8.1%) 1-year all-cause deaths. PPMI was not associated with higher 30-day (HR 1.69, 95% CI 0.38–7.50, *p* = 0.488) and 1-year all-cause mortality rate (HR 1.90, 95% CI 0.81–4.47, *p* = 0.139) (Table 7 and Fig. 2).

**Table 7:** Cox regression models for 30-day and 1-year all-cause mortality

	Univariate			Multivariate		
	HR	95% CI	*p* value	HR	95% CI	*p* value
*30-day all-cause mortality*						
EUROSCORE II	1.15	1.06–1.23	< 0.001	1.16	1.08–1.19	< 0.001
AKI	1.96	1.69–5.49	0.025	1.41	0.44–4.14	0.548
Vascular complications	2.86	0.98–8.36	0.052	1.06	0.24–4.31	0.876
Life-threatening/major bleeding complications	7.64	2.61–22.35	< 0.001	5.87	1.28–26.58	0.019
PPMI	1.69	0.38–7.50	0.488	–	–	–
*1-year all-cause mortality*						
EUROSCORE II	1.09	1.03–1.15	0.002	1.08	1.04–1.13	0.031
AKI	2.83	1.59–5.07	< 0.001	2.59	1.20–5.35	0.020
Baseline CrCl	0.97	0.95–0.99	0.002	0.97	0.96–1.01	0.092
Baseline NT-proBNP	1.01	1.01–1.02	0.003	1.00	0.99–1.01	0.540
Non-transfemoral access	1.77	1.20–3.95	0.016	1.29	0.45–3.01	0.510
Life-threatening/major bleeding complications	4.49	2.17–9.27	< 0.001	3.13	0.81–14.11	0.114
PPMI	1.90	0.81–4.47	0.139	–	–	–

*AKI* acute kidney injury, *BNP* brain natriuretic peptide, *CrCl* creatinine clearance, *PPMI* post-procedure myocardial injury

Cox regression models adjusted for age and sex showed only EUROSCORE II (HR 1.16, 95% CI 1.08–1.19, *p* < 0.001) and life-threatening/major bleeding complications (HR 5.87, 95% CI 1.28–26.58, *p* = 0.019) as independent predictors of 30-day all-cause mortality, and EUROSCORE II (HR 1.08, 95% CI 1.04–1.13, *p* = 0.031) and AKI (HR 2.59, 95% CI 1.20–5.35, *p* = 0.020) as independent predictors of 1-year all-cause mortality (Table 7).

Finally, similar results consistent to previous Cox models were observed when we repeated Cox regression analysis excluding those patients who received transapical access (Table 8).

**Table 8:** Cox regression models for 30-day and 1-year all-cause mortality excluding patients who received transapical access

	Univariate			Multivariate		
	HR	95% CI	*p* value	HR	95% CI	*p* value
*30-day all-cause mortality*						
EUROSCORE II	1.15	1.07–1.24	< 0.001	1.13	1.05–1.21	0.002
AKI	2.17	1.75–6.25	0.022	1.44	0.48–4.36	0.514
Vascular complications	3.11	1.04–9.28	0.042	1.06	0.24–4.66	0.938
Life-threatening/major bleeding complications	8.47	2.84–25.28	< 0.001	6.20	1.37–28.06	0.018
PPMI	3.47	0.45–26.53	0.230	–	–	–
*1-year all-cause mortality*						
EUROSCORE II	1.08	1.02–1.15	0.005	1.07	1.02–1.15	0.034
AKI	2.29	1.27–4.15	0.006	1.27	1.13–3.06	0.020
Baseline CrCl	0.97	0.95–0.99	0.011	0.99	0.96–1.02	0.132
Baseline NT-proBNP	1.01	1.01–1.02	0.006	1.00	0.99–1.01	0.198
Life-threatening/major bleeding complications	4.97	2.39–10.35	< 0.001	3.99	0.91–11.79	0.069
PPMI	2.19	0.86–5.55	0.098	–	–	–

*AKI* acute kidney injury, *BNP* brain natriuretic peptide, *CrCl* creatinine clearance, *PPMI* post-procedure myocardial injury

## Discussion

In this single-center, retrospective study we found that: (1) PPMI occurs in 2/3rds of patients undergoing TAVR; (2) SEV implantation is associated with a twofold higher incidence of PPMI rate as compared to BEV, even after adjusting for several a priori confounders and procedural variables; (3) PPMI does not significant impact on all-cause 30-day and 1-year mortality.

Myocardial infarction (MI) is a rare and potentially life-threatening complication of TAVR, usually caused by mechanical interference of prosthesis with coronary ostia, nonetheless a PPMI which is based on bio-humoral markers of myocardial injury is commonly reported and generally clinically silent [7–9, 14–16, 19, 21]. In our cohort, we observed a PPMI rate of 79.0% which is one the highest reported in the literature so far [6, 15, 22–24]. This result is probably explained by several factors: first and foremost, we adopted the new VARC-2 definition of myocardial injury and used the troponin I as the biomarker of choice: TnI seems to be less stable and more related to peri-procedural complications and non-cardiac cause of PPMI (e.g., ischemia related to anemia or dehydration, acute kidney injury, or severe hypoxia due to acute respiratory failure) than CK-MB [19], which was used in some previous studies [7–9]. Second, several baseline characteristics (e.g., lower average CrCl and higher NYHA class), procedural variables (such as longer procedural times) and incidence of post-procedural complications (e.g., higher significant bleedings or need for pacemaker implantation) resulted higher in ours than in other comparable cohorts, perhaps due to the inclusion of “real-world” patients across 10 years, spanning the learning curve of the center.

In agreement with previous data, we demonstrate an association between PPMI and higher LVEF, probably because viable myocardial tissue results in higher enzymes release, and also with worse baseline kidney function [8, 22–24], whereas we could not observe an independent association between PPMI and pre-TAVR valvuloplasty, mean procedural duration, and previous MI or CAD as reported in other cohorts [8, 9, 17, 22–24], probably due to heterogenous sampling and selection biases.

PPMI has a mainly procedural origin, related to mechanical trauma to the myocardium due to contact with valve struts, potential multiple episodes of hypotension during valve release, and myocardial ischemia due to balloon valvuloplasty and/or valve implantation itself [15, 17]. Of note, we found that SEV implantation might be associated to a higher risk of PPMI has already been reported, but mainly as a collateral result, and without adjustment for other procedural and clinical variables [8, 17, 22–24]. We employed a rigorous analytic approach that included multivariable and non-parsimonious propensity-weighted analysis: the most important implication of our study, thus, is that, when the choice of implanting a SEV is made, a two- to threefold higher risk of PPMI should be accounted.

The reasons for the increased PPMI risk and SEV technology are multiple: whereas in SEV implantation, the need for rapid pacing and the consequent extreme periods of hypotension are less than in BEV, SEV usually leads to peri-valvular myocardial compression (often for greater valve oversizing leading to deeper positioning of the metal frame), the release time can be longer, and there is more arrhythmic potential due to more frequent need for inotropic support [9, 10, 15, 23, 25]. All these mechanisms may lead to hypoperfusion-induced ischemia and hemodynamic instability occurring more frequently than in BEV. In addition, it could be postulated that BEV require only a brief high-pressure balloon inflation during implantation, whereas the self-expanding frame in SEV applies continuous pressure to the surrounding structures, which in turn might result in substantially greater myocardial damage, finally SEV may need multiple repositioning as compared with BEV.

We could not identify a significant relationship between PPMI and 30-day and 1-year all-cause mortality post-TAVR: the literature data are highly controversial on this topic, since several publications [7, 8, 17, 22, 26] found that PPMI is clearly linked to higher mortality, whereas other reports have been unable to find any significant association [5, 23, 24]. Nevertheless, a possible reason for this discrepancy is that we evaluated all-cause mortality, whereas PPMI was shown to be mainly associated with cardiovascular mortality [7, 8, 22, 26].

A recent meta-analysis by Michail et al. reported higher overall 30-day (5.2%) and 1-year (18.6%) all-cause mortality than our study (2.5% and 8.1%, respectively) [17]. Furthermore, most previous publications included TAVR cohorts with a significant prevalence of femoral surgical and transapical access and with substantial rates of complications such as pericardiocentesis, pericardiotomy, and conversion to open heart surgery [5, 7–9, 14, 17, 19, 21]. Since we limited our analysis to successful TAVR and there was a very low rate of serious complications in the present study, as well as only 15/596 transapical procedures, PPMI conceivably did not affect the prognosis as its association with adverse events affecting mortality was blunted.

In conclusion, the debate on the prognostic role of PPMI after TAVR and its variable link with poor outcomes is still open similarly to that of type 4A MI after coronary interventions [27–31]; in this scenario, our data support the concept that PPMI occurs quite commonly and may mainly represent a “proxy” of higher comorbidity or of peri-procedural complications.

PPMI risk is two- to threefold higher when SEV are used as compared with BEV, but it does not impact all-cause mortality. Therefore, dedicated studies with careful patients’ selection and a prospective design are needed to define the role of valve different technologies on PPMI.

## Limitations

First, we did not measure CK-MB, which is more stable than TnI and less prone to non-cardiac rise due to several acute and chronic comorbidities [19]. Second, despite we recorded the history of prior CAD, we had no information on the completeness of revascularization pre-TAVR. However, incomplete revascularization seems to not affect PPMI in previous studies [5, 6]. In addition, we have no data on aortic annulus manipulation (i.e., repositioning/retrieval of the valve) and we did not evaluate myocardial injury with more accurate imaging technique (e.g., cardiac magnetic resonance). Finally, patients were not randomly allocated to a specific valve type, albeit propensity-weighted analysis on the average treatment effect could reduce this bias.
